# The Effect of Biceps Procedure on the Outcome of Rotator Cuff Reconstruction

**DOI:** 10.1155/2013/840965

**Published:** 2013-02-13

**Authors:** Juha Kukkonen, Juho Rantakokko, Petri Virolainen, Ville Äärimaa

**Affiliations:** Department of Orthopaedics and Traumatology, Turku University Hospital, P.O. Box 28, 20701 Turku, Finland

## Abstract

*Purpose*. Biceps long head pathology is often associated with rotator cuff tears. The aim of this study was to determine the effect of possible associated biceps procedure on the treatment outcome in rotator cuff repair. *Methods*. 148 consecutive shoulders operated for isolated full-thickness supraspinatus tendon tear were included. A biceps tenotomy or tenodesis was performed in cases of irritated/frayed and/or unstable biceps tendon. The patients were grouped into three groups according to the biceps procedure (no procedure, tenotomy, and tenodesis). The age-adjusted Constant score was used as an outcome measure. *Results*. 145 shoulders (98%) were available for final followup. Preoperatively, there was no statistically significant difference in Constant scores. At three months, there was a statistically significant positive change in Constant scores compared with preoperative status in the tenotomy group in women. At one year there was a statistically significant positive change in Constant scores in all groups in both genders. However, there was no statistically significant difference between the groups at one year in either gender. *Conclusion*. Biceps procedure does not affect the final clinical treatment outcome after rotator cuff repair. Recovery from operative treatment may be faster in tenotomized female patients in cases of encountered biceps pathology.

## 1. Introduction

Biceps long head tendon (BLHT) has been thought to be a significant source of anterior shoulder pain [1]. Biceps tendon pain is often difficult to assess clinically [2]. Lafosse et al. reported that preoperative tests for biceps tendon pathology did not correlate with intraoperatively observed findings [3]. Therefore, the decision of whether to treat possible biceps pathology is often made intraoperatively during the arthroscopic evaluation.

A rupture or instability of BLHT is rarely an isolated condition and is commonly associated with rotator cuff tendon pathology [4–6]. Lafosse et al. reported BLHT instability in 45% of the patients with an operatively treated rotator cuff tear [3]. Accordingly, Chen et al. found BLHT pathology in at least 76% of the patients with an operatively treated rotator cuff tear [7]. Both biceps tenotomy and tenodesis have been reported as effective procedures to manage BLHT pathology also adjunct with rotator cuff pathology [8–11]. However, biceps tenotomy may be regarded as an anatomically, functionally, and cosmetically compromising procedure and may potentially cause harm to the patient [12, 13].

The aim of this study was to evaluate the effect of concomitant biceps tenotomy or tenodesis compared with no biceps procedure on the postoperative outcome in operatively repaired isolated supraspinatus tears. The hypothesis was that associated biceps procedure has a negative effect on the treatment outcome in rotator cuff repair compared with no procedure. 

## 2. Materials and Methods

A register (ArtuX, BCB Medical, Turku, Finland) for all patients undergoing shoulder surgery at Turku University Hospital was established in 2007. Indication for operative treatment was a clinical suspicion of a rotator cuff tear, with pain and weakness of the involved shoulder. Contraindications included stiffness, severe radiographic osteoarthritis, poor compliancy, and severe internal disease. A cohort of 148 consecutive shoulders with a repaired, isolated, full-thickness 5–25 millimeters (AP-dimension) supraspinatus tendon tear were during years 2007–2009 enrolled in this study. The patients were grouped according to the concomitant biceps procedure.

All operations were performed arthroscopically in a similar fashion by three experienced shoulder surgeons. The size of isolated supraspinatus tear was measured with an arthroscopic measuring probe after bursectomy of the subacromial space. All tendon tears were reinserted anatomically to the native foot-print with nonabsorbable titanium anchors (Corkscrew, Arthrex, Naples, FL, USA). If BLHT was noted to be irritated/frayed and/or unstable due to the rotator cuff tear, tenotomy or tenodesis was performed according to the surgeon's decision. Tenotomy was performed by cutting BLHT from the labral insertion. In tenodesis, BLHT was extra-articularly further reinserted into the revised and decorticated intertubercular groove with an individual-nonabsorbable titanium suture anchor using lasso-loop-type locking sutures (Corkscrew, Arthrex, Naples, FL, USA). Additional acromioplasty was performed in all cases. Patients were discharged from the hospital on the first postoperative day. A supporting sling was used for two weeks postoperatively. At two weeks postoperatively patients were called in for a physiotherapist's guidance for the start of passive movement exercises, and at six weeks active motion exercises were begun. Strength exercises were begun at 10 weeks after the operation. Age-adjusted Constant score was used as an outcome measure and was measured by an independent physiotherapist preoperatively, and at three months and one year after the operation. Based on previous reports [14] symptoms associated with BLHT were separately evaluated, subjectively and objectively (mean 1.6 years postoperatively, range 1–3 years) (Table 1). The results of women and men were analyzed separately.

### 2.1. Statistical Methods

The differences in categorical variables between groups were tested with chi-square test or Fisher's exact test. The differences in one-year postoperative Constant score and in the Constant change during three-month and one-year followups were analyzed by adjusting for age using linear model. Analyses were done separately for men and women. *P* values less than 0.05 were considered statistically significant. The statistical analyses were made using SAS System for Windows, release 9.2 (SAS Institute Inc., Cary, NC, USA).

## 3. Results

145 shoulders were available for one-year followup (62 women and 83 men) making the dropout rate 2%. No infectious complications were detected. There was no difference in the preoperatively detected symptoms related to possible biceps pathology between the groups. Mean age of women was 57.4 (SD 8.6) and men 57.9 (SD 10.3) years. No biceps procedure (group 0) was performed in 85 patients, 35 women (mean age 56.3 years) and 50 men (mean age 57.3 years). Tenotomy (group 1) was performed in 30 patients, 15 women (mean age 62.7 years) and 15 men (mean age 63.7 years). Tenodesis (group 2) was performed in 30 patients, 12 women (mean age 54.1 years) and 18 men (mean age 54.9 years). The difference in ages between the groups was not statistically significant in either gender (*P* = 0.5757, *P* = 0.0716).

Preoperatively, there was no statistically significant difference in Constant scores between the groups in either gender (*P* = 0.2104, *P* = 0.3475).

At three months, there was a statistically significant positive change in Constant scores in women compared with preoperative status in group 0 and group 1. However there was no such improvement in group 2. In men there was a statistically significant positive change in Constant scores compared with preoperative status in group 0. The improvement in Constant scores was not statistically significant in group 1 and group 2 (Table 2).

**Figure 1:**
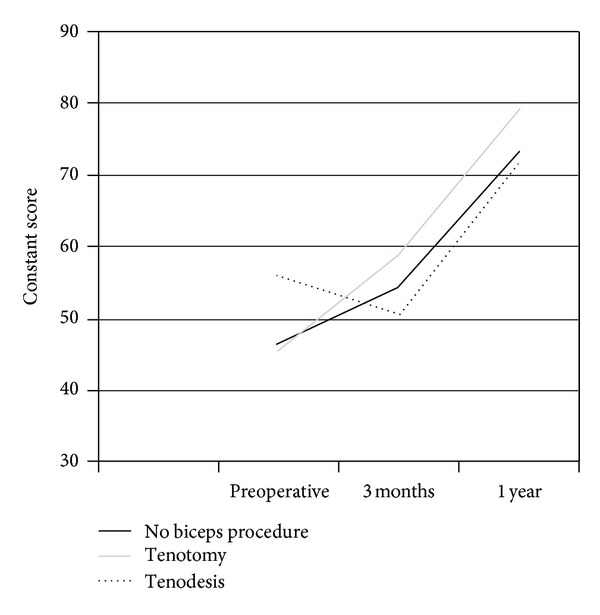
Constant scores in women.

**Figure 2:**
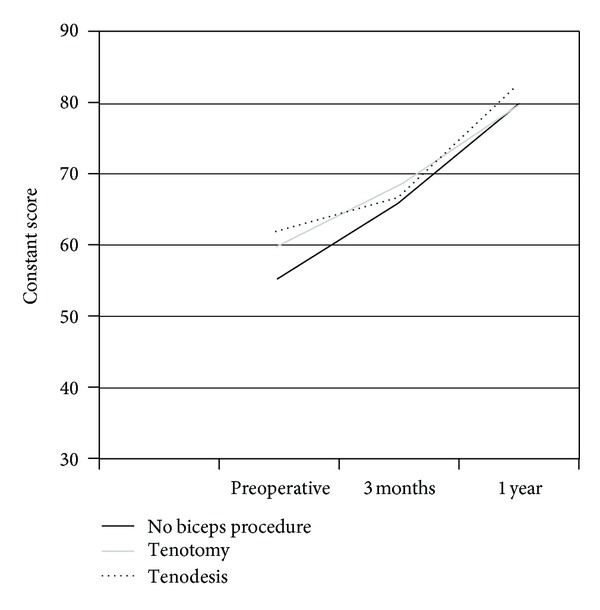
Constant scores in men.

At one year, there was a statistically significant positive change in Constant scores compared with preoperative status in all groups in both genders. In women, the best scores were in group 1, but there was no statistically significant difference between the groups at one-year followup. There was no statistically significant difference between the groups at one year in men (Table 2) (Figures 1 and 2).

26 (87%) of shoulders with BLHT tenotomy and 24 (80%) of shoulders with tenodesis were available for separate study visit specifying the symptoms related to BLHT and biceps muscle. A Popeye deformity occurred in 8 (31%) in the tenotomy group and in 3 (13%) in the tenodesis group. This difference was statistically significant in men. Fatigue or subjective weakness of biceps muscle was found in 1 (4%) patient in both groups. Occasional arm cramping was reported by 2 (8%) patients in the tenotomy group. In the tenodesis group, none of patients reported this symptom. These subjective symptoms did not show statistically significant difference in either gender (Table 1).

## 4. Discussion

In our study, there were no significant differences in preoperative Constant scores between the groups. The indication for operative treatment was clinical suspicion of supraspinatus tear only and no difference was detected between the groups regarding the possible biceps pathology. Furthermore, intraoperatively detected BLHT pathology did not affect the preoperative scores reflecting the previously described difficulty in detecting BLHT pathology clinically. The mean improvement of Constant scores at three months was greater in the tenotomy group than in the tenodesis group. This finding was statistically significant in women. Therefore, postoperative recovery may be faster in the tenotomy group as compared to the tenodesis group in cases of associated biceps pathology. At one year, there was no statistical significant difference in Constant scores between the groups in either gender.

In our material, the postoperative Popeye deformity was more common in the tenotomy group. However, the Popeye deformity did not occur in 69% of the patients in the tenotomy group. It is also noteworthy that 13% of the patients in the tenodesis group developed the Popeye deformity. It has been suggested that inflammation in the rotator interval, BLHT, or bicipital groove may result in an autotenodesis effect which diminishes the prevalence of the Popeye deformity [14]. In a previous study, despite the Popeye deformity, no other differences between tenotomy and tenodesis in conjunction with rotator cuff repair were reported [14]. The Popeye deformity has been suggested to cause arm cramps and pain manifestation also after tenodesis procedure [15]. In our study, the Popeye deformity was not related to symptoms of the biceps muscle and only two patients in the tenotomy group reported occasional arm cramps at one-year followup. Accordingly, it has been previously reported that there is no difference in subjective symptoms in the same-aged patients comparing to these two biceps procedures [14].

Constant scoring is endorsed by SECEC/ESSSE and is the most largely used instrument measuring shoulder function. This scoring system is very suited for evaluating rotator cuff ruptures [16]. It has been reported that after arthroscopic rotator cuff repair the Constant score significantly improves until one year, after which it will be stabilized [17]. The Constant score has been noted to be age- and sex-related [18]. Furthermore, the size of the rotator cuff tear is known to affect Constant scores [19]. We wanted to minimize the confounding factors and included only small/medium-size, full-thickness, isolated supraspinatus tears in our cohort. Furthermore, we used age-adjusted Constant scores and analyzed the results of women and men separately. The scores were analyzed and presented separately in women and men, as there may be clinical assumptions that men do not tolerate the tenotomy procedure as well as women [12]. The standardized setup with prospectively gathered register information is a strength of this study.

Due to a retrospective register study setup, no power analysis was performed. Because of rather small number of patients in each group, the results must be interpreted with caution. 60 (41%) of the included patients had intraoperatively detected biceps pathology and underwent a concomitant biceps procedure. This is in accordance with previous reports of associated biceps pathology [3, 5]. The indication and type of the performed biceps procedure was the operator's subjective view on the BLHT pathology. This is a clear weakness of this study, although all the involved surgeons performed the procedures in a similar fashion. In order to find out the effect of BLHT procedures compared to no procedure, an exact objective description of the BLHT pathology together with a randomized setup would be needed. However, a comparison between tenotomy and tenodesis in our study may be performed as both procedures had the same indication and structural findings. MRI investigation could possibly help to objectively evaluate the condition of BLHT and its surrounding structures. The lack of systematic preoperative MRI, may, therefore be regarded as a weakness; however, pathological changes in BLHT may not always be visible on MRI [20]. Postoperative MRI would also help to evaluate the rotator cuff. Nho et al. reported that concomitant biceps procedure was associated with a tendon defect after arthroscopic rotator cuff repair. They found that in cases with concomitant biceps tenotomy or tenodesis there was 11 times the risk of a postoperative rotator cuff defect compared with cases without a biceps procedure [21]. Due to the lack of postoperative MRI investigations, we do not know the prevalence of supraspinatus rerupture in our groups. However, Constant scores improved in all groups, and there was no statistical significant difference in scores between the groups at one year.

The role of BLHT as a pain generator in the shoulder has been emphasized in many studies [1, 22]; hence, both tenotomy and tenodesis are universally recommended treatment options for BLHT pathology [5, 11, 12, 23]. As an isolated procedure, the advantages of tenotomy over tenodesis are simplicity, short surgical time, low surgical morbidity, avoidance of implant complication, and simple rehabilitation [24, 25]. However, when BLHT procedure is associated with an arthroscopic rotator cuff repair the meaning of abovementioned advantages diminish and there is still debate about optional BLHT procedure in these cases. In the literature, there are no prospective, randomized studies comparing BLHT tenotomy and tenodesis in the setting of arthroscopic rotator cuff repair.

## 5. Conclusion

This study showed that BLHT procedure did not impair the final clinical treatment outcome as compared to no procedure. Furthermore, recovery evaluated as improvement of Constant score may be faster in tenotomized women compared to tenodesis in cases of encountered biceps pathology in association with supraspinatus tear. A nonanatomic BLHT tenotomy does not seem to cause any significant disadvantages for the patients. Further sufficiently numbered prospective studies are needed to investigate the differences between tenotomy and tenodesis in association with rotator cuff repair and also in conjunction with other types of rotator cuff tears.

## Figures and Tables

**Table 1:** Evaluated symptoms associated to biceps muscle in the tenotomy versus tenodesis group in women and men.

	Tenotomy		Tenodesis		
Symptom	*n* = 26		*n* = 24		
	♀ 13	♂ 13	♀ 9	♂ 15	
Popeye deformity	*n* = 8 (31%)		*n* = 3 (13%)		*P* = 0.1192
Women	*n* = 0 (0%)		*n* = 0 (0%)		*P* = 1.0000
Men	*n* = 8 (62%)		*n* = 3 (20%)		*P* = 0.0248
Fatigue or subjective weakness of biceps muscle	*n* = 1 (4%)		*n* = 1 (4%)		*P* = 1.0000
Women	*n* = 1 (8%)		*n* = 0 (0%)		*P* = 0.3944
Men	*n* = 0 (0%)		*n* = 1 (7%)		*P* = 0.3431
Arm cramping	*n* = 2 (8%)		*n* = 0 (0%)		*P* = 0.4906
Women	*n* = 2 (15%)		*n* = 0 (0%)		*P* = 0.4935
Men	*n* = 0 (0%)		*n* = 0 (0%)		*P* = 1.0000

**Table 2:** Constant scores in women and men (no biceps procedure, tenotomy and tenodesis).

Preoperative and 1-year postoperative age-adjusted Constant scores				
	♀	♀	♂	♂

No biceps procedure	46.6 (SE 2.8)	73.1 (SE 1.8)	55.4 (SE 2.4)	79.6 (SE 1.9)
Tenotomy	45.4 (SE 4.4)	79.2 (SE 2.8)	60.0 (SE 4.7)	79.5 (SE 3.7)
Tenodesis	55.9 (SE 4.8)	71.7 (SE 3.0)	61.8 (SE 4.1)	82.5 (SE 3.2)
	*P* = 0.2104	*P* = 0.1328	*P* = 0.3475	*P* = 0.7178

Improvement of Constant scores at 3 months compared to preoperative scores				
	♀	♂		

No biceps procedure	7.8 (*P* = 0.0257)	10.9 (*P* < 0.0001)		
Tenotomy	13.6 (*P* = 0.0134)	8.7 (*P* = 0.0766)		
Tenodesis	−5.1 (*P* = 0.3828)	5.2 (*P* = 0.2017)		

Improvement of Constant scores at 1 year compared to preoperative scores				
	♀	♂		

No biceps procedure	26.5 (*P* < 0.0001)	24.1 (*P* < 0.0001)		
Tenotomy	33.8 (*P* < 0.0001)	19.5 (*P* < 0.0001)		
Tenodesis	15.7 (*P* = 0.0037)	20.7 (*P* < 0.0001)		

SE: Standard Error.
